# Preliminary clinic study on computer assisted mandibular reconstruction: the positive role of surgical navigation technique

**DOI:** 10.1186/s40902-015-0017-1

**Published:** 2015-07-30

**Authors:** Jin-Wei Huang, Xiao-Feng Shan, Xu-Guang Lu, Zhi-Gang Cai

**Affiliations:** Department of Oral and Maxillofacial Surgery, Peking University School and Hospital of Stomatology, 22# Zhongguancun South Avenue, Haidian District 100081 Beijing, China

**Keywords:** Computer assisted surgery, Mandibular defect, Fibula flap, Surgical navigation

## Abstract

**Background:**

The objectives of the present study were to investigate the reliability and outcomes of computer-assisted techniques in mandibular reconstruction with a fibula flap and verify whether the surgical navigation system was feasible in mandible reconstructive surgery.

**Methods:**

Eight cases were enrolled in the computer assisted surgery (CAS) group and 14 cases in the traditional group. The shaping and fixation of the fibula grafts were guided by computer assisted techniques, which could be monitored with the BrainLAB surgical navigation system. The variation of mandible configuration was evaluated by CT measurement in the Mimics software, including the variation of length, width, height and gonial angle of the mandible. The 3D facial soft tissue alteration was also analyzed in 3D chromatogram by Geomagic software.

**Results:**

All 22 fibula flaps survived. The mandibular configurations and facial contours had a better clinic result in the CAS group. The length, width, height and gonial angle of the reconstructive mandible were more similar to the original one. The Wilcoxon rank sum test analysis suggested significant differences in the measurements. The chromatographic analysis also visually showed superiority over the traditional group.

**Conclusions:**

The computer assisted surgical navigation method used in mandibular reconstruction is feasible and precise for clinical application. The contour of the reconstructed mandible and facial symmetry are improved with computer techniques.

## Background

The loss of mandibular continuity caused by ablative tumor therapy, osteomyelitis or severe trauma could affect a patient’s quality of life both physiologically and psychologically. The complexity of mandibular anatomy creates a great challenge for plastic surgeons to reconstruct the facial contour and rehabilitate the occlusal function [1, 2]. With the development of surgical techniques and instruments, functional and aesthetic rehabilitation of mandibular defects have become a basic goal for surgeons [3–5].

To achieve satisfactory mandibular contour and masticatory function, the correct occlusal relationship and condyle position are particularly important. Optimal 3D configuration of the graft is the crucial factor affecting the facial contour and the occlusal relationship [6, 7]. The computer assisted surgery (CAS) techniques,which mainly consist of computer aided design/computer aided manufacture (CAD/CAM), rapid prototyping (RP), reverse engineering (RE), and surgical navigation technique, have dramatically improved the precision of graft placements. With preoperative planning and intra-operative navigation, these techniques can help plastic surgeons to manage mandibular reconstructive surgery. This study compared the CAS group and the traditional group of mandibular reconstruction using free fibula flaps to analyze the variations of the mandible and 3D facial contour.

## Methods

Eight patients, two males and six females, with an average age of 33.3 years (24 ~ 53 years), were enrolled in the CAS group in the Oral and Maxillofacial Surgery Department of Peking University School of Stomatology, from June 2009 to June 2012. The causes of the mandibular defects were tumors, six malignant and two benign [Table 1]. The control group, as a traditional treatment, involved 14 patients during the same time period. The mean age was 54.9 years (18 ~ 78 years), seven males and seven females. All of these patients underwent mandibular reconstruction by experienced surgeons without computer assisted techniques. The operations in this study were performed by the same team of surgeons. For each patient, there would be both ablative and reconstructive surgery groups with 2–3 surgeons operating simultaneously. All of the operations were directed by one first surgeon, who has been working as a constructive surgeon for more than 10–15 years. This study had been approved by the Ethics Committee of Peking University School and Hospital of Stomatology(NO.: PKUSSIRB-201310110). Consent forms were signed by the patients and family members.

**Table 1:** Basic information of the computer assisted group

Patient	Gender	Age	Pathology	Resection area	Fibula segments	Computer technique
1	F	27	Sarcoma	Body, symphysis	3	Surgical template
2	F	48	Ossifying fibroma	Body	1	Navigation
3	F	29	Osteosarcoma	Body, ramus	2	Navigation
4	F	53	Odontogenic keratocyst	Body	1	Navigation
5	F	26	Enameloblastoma	Body, symphysis	1	Navigation
6	F	34	Osteosarcoma	Body, ramus, condyle	2	Navigation
7	M	25	Hemangiosarcoma	Body, ramus, condyle	2	Surgical template + navigation
8	M	24	Osteosarcoma	Body, symphysis	3	Surgical template + navigation

All patients received pre- and post-operative CT scans (BrightSpeed Edge Select 8 slice, USA). The medical data was recorded in a generic DICOM format and transferred to the computer work station. The 3D facial scans were also recorded by 3dMD patient Software 4.0, USA.

Before surgery, simultaneous mandibulectomy and reconstruction planning was performed in a virtual environment. The software used for virtual planning consisted of SurgiCase CMF 5.0 (Materialise,Belgium), and Mimics 10.01 (Materialise,Belgium). The surgical navigation system utilized for intra-operative supervision was the BrainLAB VectorVision iPlan® CMF 3.0 System. Geomagic Studio 12.0 software was utilized for chromatographic analysis. With upgraded point-cloud handling and polygon mesh processing, it could visualize the differences of the pre and post 3D facial configurations, used for demonstration to the patients.

### Mandibular reconstruction guided by surgical template

The reconstructed mandibular models were fabricated with the CAD/CAM technique. A titanium template was made according to the virtual design for the bone graft through rapid prototyping (RP) technique and served as a guide when surgeons were shaping and placing the fibula grafts.

### Mandibular reconstruction guided by surgical navigation technique

**Fig. 1:**
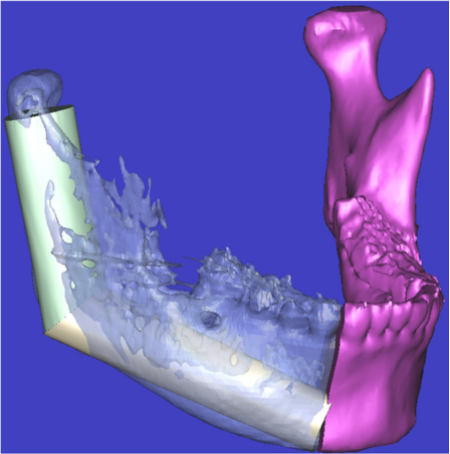
Osteosarcoma of the right condyle, ramus andmandible body. Virtual osteotomy and fibula reconstruction were simulated on the Surgicase workstation

**Fig. 2:**
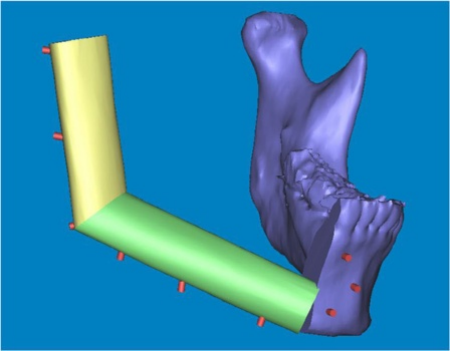
Guide points (red) were set up in the Mimics workstation, which could induce the precise placement of the fibula flap. These landmarks would be drilled on the surface of the residual mandible before osteotomy, and the reposition of these landmarks under surgical navigation suggest the original position of the residual mandible

An individual dental splint was fabricated to fix the mandible to the maxilla before surgery, which could secure the mandible as a stable part to the skull. In that case, the mobile mandible could be assumed as one rigid piece of the skull during surgical navigation. The thin-cut axial CT scan of the mandible was performed with the splint fixing the occlusion. The CT data was imported into the SurgiCase CMF 5.0, where 3D virtual osteotomies, bony reductions and fibula reconstructions were performed [Fig. 1]. The osteotomy lines and the position of the fibula graft were designed according to the 3D images in the virtual platform. Several reference landmarks were set on the residual mandible surface, along the posterior and inferior margins of the fibula as guidance for placing bone grafts [Fig. 2].

### Mandibular reconstruction guided by both the surgical template and navigation techniques

**Fig. 3:**
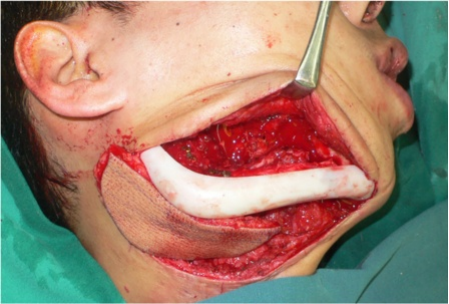
Specific fibula resin surgical template was fabricated to shape the graft, which also helped to place the fibula into the designed position

**Fig. 4:**
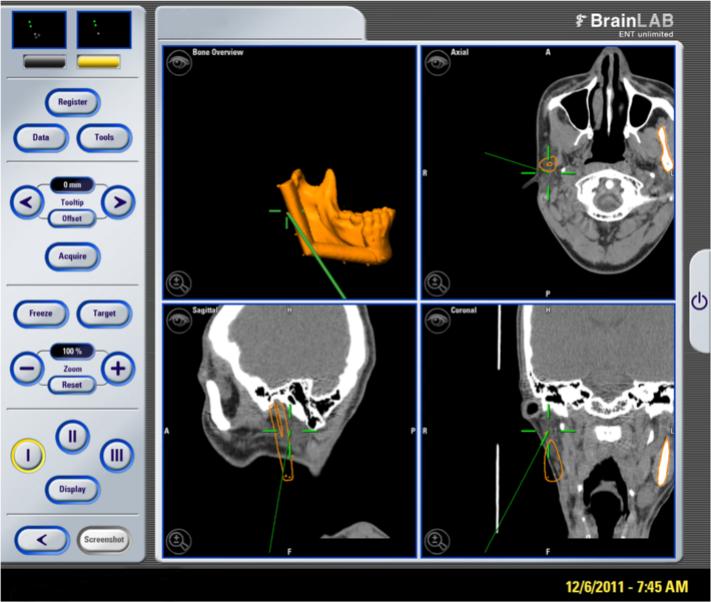
After natural head posture registration, the BrainLab navigation system permitted 3D analysis to help the surgeon to quantify a complex position of the bone graft. The navigation system projected the location of the surgical probe on a computer monitor in axial, coronal, sagittal and 3D views. The position of the fibula could be verified intra-operatively

According to the experience of the authors, in some complex cases, especially secondary reconstruction cases, a combination of the surgical template and navigation technique might improve the precision of the surgery. The surgical template was placed on the dissociated mandible to shape the graft [Fig. 3], meanwhile the residual mandible was rigidly fixed to the patient’s skull. With the help of the navigation system, the position of the grafts could be adjusted in 3 dimensions [Fig. 4].

### Outcome assessment and statistical analysis

The primary outcome measurements are the 3D dimensional changes of the mandible before and after surgery. We aimed at recreating the original dimensions of the mandible with the free fibula flaps. Therefore, an ideal outcome was that there were no changes in the 3D measurement of the distances among the selected mandibular landmarks [Table 2]. The group that had less change in 3D dimension was considered to have favorable outcomes.

**Table 2:** Mandible anatomic landmarks and 3D measurements

Abbreviation	Anatomic point	Measured item/mm
Ci	condylion internale	Ci-Ci
Cl	condylion laterale	Cl-Cl
Co	condylion	Co-Gn
Cp	coronoid process	Co-Go
Ga	gonial angle	Cp-Cp
Gn	gnathion	Go-Gn
Go	gonion	Go-Go
Lm	lingula mandibulae	Lm-Lm
Me	menton	
Po	pogonion	

**Fig. 5:**
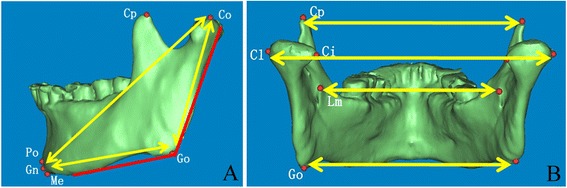
Mandible anatomic landmarks and 3D measurements. The landmarks were shown in (**a**) lateral and (**b**) posterior view

We compared the 3D changes of the mandibular configurations before and after surgery in both groups. The reference points were calculated on the Mimics software for 3 times and the average was chosen as the compared value. Anatomic landmarks indicating the length, width, height and the angle of both the deformed mandible and the reconstructive mandible were measured [Fig. 5]. For the reconstructive fibula, Ci (condylion internale) was defined as the internal vertex of the top of the fibula bone, Cl (condylion laterale) the lateral vertex and Co (condylion), the center of the top. The Gonial angle was defined as the angle of the posterior central border of the ramus and lower central border of the mandible body. Other reference points were defined in the same way as anatomy on cephalography. Among the ramus and condyle involved reconstruction, the Lm (lingula mandibulae) and CP (coronoid process) might not be able to be recovered with fibula flap, which we did not take into calculation. All measurements were performed in 3D software Mimics 10.01.

Due to the low sample size, the non-parametric analysis, Wilcoxon rank sum test was used to compare the variations of the CAS group and the traditional group, with statistical significance set at *p* < 0.05.

## Results

**Fig. 6:**
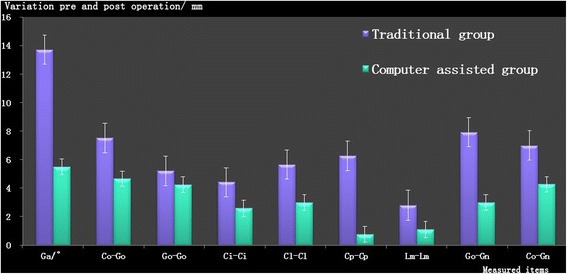
The variations of the 3D measurements

Mandibular reconstruction was performed accurately according to the virtual planning, all 22 fibula flaps survived. The anatomic landmark measurements of the mandible configuration, included the Gonial angle (Ga), the distance of Condylion - Gonion (Co-Go), Gonion - Gonion (Go-Go), Condylion internale - Condylion internale (Ci-Ci), Condylion laterale - Condylion laterale (Cl-Cl), Coronoid process - Coronoid process (Cp-Cp), Lingula mandibulae - Lingula mandibulae (Lm-Lm), Condylion - Gnathion (Co-Gn) and Gonion - Gnathion (Go-Gn), all these items in the CAS group had less change as compared to the traditional group, even though the amplitude varied respectively [Fig. 6].

Based on the Wilcoxon rank sum test analysis, the variations of Ci-Ci, Cl-Cl, Cp-Cp and Go-Gn had significant differences statistically, while Ga, Co-Go, Go-Go, Lm-Lm and Co-Gn did not [Table 3]. This result indicated that the CAS group might have advantages in maintaining the original configuration of the mandible compared to the traditional group.

**Table 3:** The statistical analysis results

Items/ |X| ± 2SE	Traditional group	CAS group	*P*
Ga/°	13.72 ± 5.77	5.49 ± 3.75	>0.05
Co-Go/mm	7.53 ± 4.47	4.65 ± 5.18	>0.05
Go-Go/mm	5.22 ± 2.40	4.24 ± 3.73	>0.05
Ci-Ci/mm	4.42 ± 1.70	2.58 ± 3.36	<0.05
Cl-Cl/mm	5.66 ± 3.45	2.99 ± 3.86	<0.05
Cp-Cp/mm	6.28 ± 2.17	0.77 ± 0.74	<0.05
Lm-Lm/mm	2.78 ± 2.73	1.09 ± 0.84	>0.05
Go-Gn/mm	7.93 ± 3.29	2.99 ± 1.93	<0.05
Co-Gn/mm	6.99 ± 3.49	4.29 ± 1.81	>0.05

**Fig. 7:**
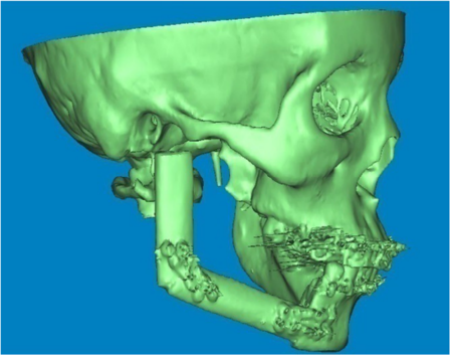
Mandible defect and fibula flap reconstruction with CAS technique

**Fig. 8:**
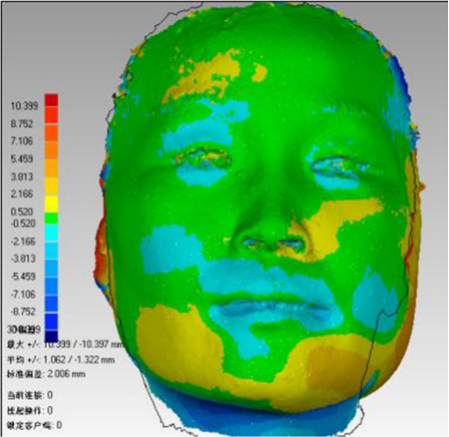
Chromatographic analysis showed satisfied outcome of the reconstructive contour

The clinical outcome showed that the configuration of the mandible was precisely recovered as compared to the original shape. The chromatographic analysis showed only a slight change of the facial contour2–4 weeks after surgery, which could exclude post-surgical swelling [Figs. 7 and 8, same case as Figs. 1 and 2].

## Discussion

Over the past 30 years, the CAS techniques have been widely used in surgical procedures worldwide. In oral and maxillofacial surgery, more and more attention has been paid to the individual and functional reconstruction of the maxillofacial defects. The concept of virtual surgery with surgical simulation rather than relying exclusively on intra-operative manual reconstruction has been widely accepted. It’s believed that particularly in complex cases, CAS techniques will improve the surgical outcomes in maxillofacial reconstruction surgery [8–11]. The optimal outcome for mandibular reconstruction is to achieve a mandible of correct shape according to the original or natural condition and satisfactory positioning of the condyle in the glenoid fossa [12–14]. The reconstruction of mandibular defects is challenging because of its complexity and unique characteristics, especially concerning facial esthetics and occlusal relations [15, 16].

With modern CAS techniques, the individual mandibular model can be fabricated using CT data, which is valuable for the shaping procedure of the bone graft in a virtual environment before the actual surgery [17–20]. Intra-operative navigation systems, initially developed for neurosurgical applications, allow the surgeons to determine the precise location of any instrument or bony anatomic landmark to within approximately 1 – 2 mm. The availability of 3D computer planning software and the use of 3D models in various surgical disciplines allows for an improved and more predictable reconstruction outcome [21–25].

The CAS techniques can enable the clinicians to operate virtually before the surgery, and progress from simple 2D images to sophisticated 3D surgical simulation covering intra-operative procedures such as virtual reality osteotomies and placement of bone grafts. Utilizing CT guided 3D techniques, an intra-operative template can be fabricated to transfer the virtual design to reality. Traditionally, the position of bone grafts are assured by the surgeons’ experience and the final result confirmed by CT scan. If any mistake occurred, there was no chance for modification besides secondary surgery. With the help of the surgical navigation system, the position of the bone graft, the dental occlusion and the condyle’s position could be confirmed intra-operatively. The surgical simulation with 3D stereolithographic models can also help to establish confidence for the clinician, improve young surgeons’ operating skills, and demonstrate the procedure for patients.

The fabrication of the 3D stereolithographic model, the usage of the dental splint, and the registration of the navigation system would require extra work before surgery. And it is debatable that whether the slightly better result is worth all of the extra preparation, as well as the increased cost to the patients. Clinicians were also questioning whether the CAS techniques require wider resection margins. In our study, depending on the malignancy of the biopsy, the resection margins were placed 5–10 mm extensively beyond the visualized borders of the lesion in 3D view. For margin safety, in some cases, wider resection might be necessary. However, since most patients enrolled would have more than 10.0 cm mandibular defects, it is considered acceptable to have wider resections.

In our study, it seemed that young age patients would prefer CAS techniques more likely than old age patients, since they were more concerned about the esthetic outcome of the reconstructive configuration. Meanwhile, young age patients tended to have more stable occlusal relation of the jaws due to less missing teeth, which would contribute to the accuracy of the mandible surgical navigation.

Registration technique is the key element in the precision of surgical navigation. People used to believe that the navigation system is not suitable for mandible surgery because of the movement of the jaw. In our study, we fabricated a dental splint to fix the mandible to the skull to control the mobility. We drilled several landmarks on the residual mandible surface before osteotomy as the reference points. After osteotomy, the recovery of the condyle and coronoid process was based on the reposition of these landmarks.

The results of our study showed that some measured items of mandibular configuration were even better in the traditional group than the CAS group. It seemed that even though the experienced surgeons can place the fibula flap in an ideal position without computer techniques, this would not be an argument against CAS techniques. Because the precise position of the fibula graft could mostly be achieved with CAS techniques while it costs a long time and effort to become an experienced surgeon, which would take at least 10 to 15 years.

## Conclusions

The new designed surgical navigation method applied in mandibular reconstruction is feasible, precise enough for clinical application. The contour of the reconstructed mandible of the computer assisted group has a better outcome than traditional group.

## Abbreviations

CAS: Computer assisted surgery
Ci: Condylion internale
Cl: Condylion laterale
Co: Condylion
Cp: Coronoid process
Ga: Gonial angle
Gn: Gnathion
Go: Gonion
Lm: Lingula mandibulae
Me: Menton
Po: Pogonion
